# Association between Hemoglobin Levels and Colorectal Polyps in Asymptomatic Chinese Adults

**DOI:** 10.1155/2020/3808163

**Published:** 2020-01-18

**Authors:** Chao Shen, Xiaoying Shi, Maram Al-Ashoor, Chengfu Xu

**Affiliations:** ^1^Health Management Center, The First Affiliated Hospital, College of Medicine, Zhejiang University, Hangzhou 310003, China; ^2^Department of Gastroenterology, The First Affiliated Hospital, College of Medicine, Zhejiang University, Hangzhou 310003, China

## Abstract

**Methods:**

A cross-sectional analysis was performed among 1633 asymptomatic adults who underwent colonoscopy examinations during routine health check-ups at the First Affiliated Hospital, College of Medicine, Zhejiang University, between 2015 and 2018.

**Results:**

A total of 449 (27.50%) participants were diagnosed with colorectal polyps, and those with colorectal polyps had significantly higher hemoglobin levels than did those without colorectal polyps. Hemoglobin levels were positively associated with the prevalence of colorectal polyps, with rates of 16.43%, 26.20%, 32.17%, and 35.87% among participants with hemoglobin levels in the first, second, third, and fourth quartiles, respectively (*P* for trend < 0.001). Stepwise multiple regression analysis showed that elevated hemoglobin levels independently increased the risk of colorectal polyps (odd ratio = 1.017; 95% confidence interval: 1.008–1.026).

**Conclusion:**

Elevated hemoglobin levels were significantly and independently associated with the prevalence and risk of colorectal polyps in asymptomatic adults.

## 1. Introduction

Colorectal cancer (CRC) is ranked as the second most common cancer diagnosed in women and the third most common cancer in men worldwide [1]. The incidence and mortality of CRC have shown an increasing trend in China over the past decade [2]. This trend can be attributed to many factors, including rapid urbanization, an aging population, and a shift toward a more sedentary lifestyle and unhealthy dietary habits [2].

It has been well established that most CRCs develop from adenomatous polyps. The pathogenesis of CRC has been extensively studied, and most invasive CRCs develop from adenomas following the adenoma-carcinoma sequence [3]. In general, CRCs that arise sporadically result from the stepwise accumulation of multiple somatic mutations [4]. This hypothesis is supported by pathological, epidemiological, and observational clinical data, and it is widely accepted that early detection and resection of these polyps may prevent CRC-related death [5]. Multiple CRC screening guidelines have been issued. Although recommendations vary, they are commonly based on the individual's age and risk factors [6]. However, due to certain limitations, such as the availability of national screening programs and medical resources, CRC screening in China is less likely to be conducted in the average-risk population [7]. Identifying risk factors and associations of certain inexpensive laboratory parameters can be useful in targeting groups of people for colonoscopy who are generally regarded as average-risk individuals.

A simple blood test, which is normally ordered during an annual routine health screening, can detect certain aberrations in laboratory parameters. These aberrations may be utilized to help physicians identify and stratify populations with high incidences of colorectal polyps or CRC. Kinar et al. reported that the information contained in the complete blood count report could be processed automatically by a machine learning flagging system to identify individuals with high risk of CRC [8]. In addition, hemoglobin levels have been shown to be elevated in metabolic diseases that are closely associated with colorectal polyps, such as metabolic syndrome and nonalcoholic fatty liver disease [9–12]. Chung et al. reported that an elevated hemoglobin level is associated with an increased risk of developing metabolic syndrome [9]. Our previous study, along with other studies, also found that hemoglobin levels are positively associated with both the prevalence and incidence of nonalcoholic fatty liver disease [10, 11], and elevated glycosylated hemoglobin levels are useful in predicting adenomatous polyps [13]. These hematologic and metabolic parameters can readily be obtained through inexpensive routine screening tests, and identifying associations between certain laboratory parameters and the risk of colorectal polyps may be of great use in the early detection of colorectal polyps.

In this study, we aimed to investigate whether hemoglobin levels are associated with the prevalence and risk of colorectal polyps in average-risk asymptomatic adults.

## 2. Materials

### 2.1. Study Population

This cross-sectional study was performed among asymptomatic adults who underwent colonoscopy examinations during routine health check-ups at the First Affiliated Hospital, College of Medicine, Zhejiang University, between 2015 and 2018. Because we mainly focused on the average-risk screening population, the following participants were excluded: those with a personal history of colonic diseases (colorectal cancer, colorectal polyps, and inflammatory bowel disease), a history of colorectal surgery, or a family history of colorectal cancer or hereditary cancer syndrome. We also excluded patients who underwent colonoscopies that did not reach the cecum and those with inadequate bowel preparation (Boston Bowel Preparation Scale score less than six). A total of 1633 participants (1123 men and 510 women) were enrolled in this study. The study protocol was approved by the hospital ethics committee.

### 2.2. Clinical Evaluations

Trained physicians performed the clinical evaluations according to standardized procedures [14, 15]. Standing height, waist circumference, and body weight without shoes and with light clothes were recorded in the morning using standard procedures. The body mass index (BMI) was calculated as the body weight (kg) divided by the height squared (meters). Blood pressure was measured in the sitting position after 10 min of rest using an automated sphygmomanometer following a standard protocol.

A fasting blood sample was obtained from each participant for analysis of blood hemoglobin level and serum biochemical variables. The blood hemoglobin level was analyzed using an automated hematology analyzer (Sysmex XT-1800, Kobe, Japan) according to the manufacturer's instructions. Serum biochemical variables were measured using a Hitachi 7600 autoanalyzer (Hitachi, Tokyo, Japan) with standard methods.

### 2.3. Colonoscopy Examination

Experienced endoscopists performed colonoscopies after the bowel was adequately prepared. The preparation included self-administration of 2-4 L of polyethylene glycol solution six hours before the colonoscopy. The endoscopists determined the adequacy of the bowel preparation using the Boston Bowel Preparation Scale (BBPS). A successful colonoscopy was defined as one with cecal intubation, which was confirmed by photography. The size, number, and location of polyps were recorded by the endoscopists for further analysis. Those with a successful colonoscopy and a BBPS score of six or higher were included in the final analysis.

### 2.4. Statistical Analysis

Statistical analyses were performed using SPSS 18.0 software for Windows (SPSS Inc., Chicago, IL). Quantitative variables are expressed as the mean and standard deviation for normally distributed variables or median and interquartile range for nonnormally distributed variables. Student's *t*-tests, Mann-Whitney *U* tests, or chi-square tests were applied to compare variables. Multiple stepwise regression analysis (backward: Wald; cutoff for entry: 0.05; cutoff for removal: 0.10) was performed to explore risk factors for colorectal polyps. *P* values less than 0.05 (2-tailed) were considered to indicate statistical significance.

## 3. Results

### 3.1. Hemoglobin Levels Are Elevated in Participants with Colorectal Polyps

Among the 1633 participants included in the analysis of this study, 449 (27.50%) were diagnosed with colorectal polyps; three participants were found to have histologically confirmed colorectal carcinoma. Among those with polyps, 267 (59.47%) participants had a single polyp and 182 (40.53%) had two or more. Most of the participants (*n* = 300, 66.82%) had left-sided polyps, 97 (21.60%) had right-sided polyps, and 52 (11.58%) had both-sided polyps. The size of polyps ranged from 2.0 mm to 40.0 mm with a medium (interquartile range) of 4.0 mm (2.0–6.0 mm). For 232 participants, biopsies or resections of colorectal polyps were performed; 139 (59.91%) were identified as having neoplastic polyps and 93 (40.09%) as having nonneoplastic polyps. Clinical characteristics were compared between those with and those without colorectal polyps (Table 1).

The participants with colorectal polyps were older, predominantly male, and had a greater waist circumference, a higher BMI, and a higher blood pressure than those without colorectal polyps (Table 1). Those with colorectal polyps also had significantly higher serum levels of alanine aminotransferase (ALT), aspartate aminotransferase (AST), *γ*-glutamyltransferase (GGT), triglycerides, uric acid, and carcinoembryonic antigen (CEA), as well as higher total and LDL cholesterol and fasting blood glucose, than those without colorectal polyps. In addition, serum HDL cholesterol levels in participants with colorectal polyps were lower than those in participants without colorectal polyps (all with *P* < 0.01, Table 1). These results indicate that participants with colorectal polyps had worse metabolic profiles than those without colorectal polyps. Another interesting finding is that hemoglobin levels were significantly higher in participants with colorectal polyps than in those without colorectal polyps (Table 1). We also found that hemoglobin levels tended to be higher in participants with neoplastic polyps than in those with nonneoplastic polyps (152.3 ± 13.3 g/L vs. 149.3 ± 15.2 g/L, *P* = 0.111). These findings indicated that hemoglobin levels are potentially associated with colorectal polyps, and the levels may have a stronger association with neoplastic polyps than with nonneoplastic polyps.

### 3.2. Hemoglobin Levels Are Positively Associated with the Prevalence of Colorectal Polyps

To further explore the association between hemoglobin level and colorectal polyps, we divided all participants into quartiles according to their hemoglobin level: quartile 1, hemoglobin ≤ 136 g/L; quartile 2, 136 g/L < hemoglobin ≤ 150 g/L; quartile 3, 150 g/L < hemoglobin ≤ 159 g/L; and quartile 4, hemoglobin > 159 g/L. We observed the hemoglobin quartiles to be positively correlated with the prevalence of colorectal polyps. The prevalence of colorectal polyps was 16.43% among the participants with hemoglobin levels in the first quartile, and the prevalence increased to 26.20%, 32.17%, and 35.87% in the second, third, and fourth quartiles, respectively (*P* for trend < 0.001, Table 2). This finding shows that individuals who have higher hemoglobin levels are more likely to have colorectal polyps.

### 3.3. Hemoglobin Levels Are Associated with Colorectal Polyps Independent of Obesity

Obesity is a major risk factor for CRC and a potential cofactor for the association between hemoglobin level and colorectal polyps. To explore whether this association is independent of obesity, we excluded 680 participants who were overweight/obese (BMI ≥ 25 kg/m^2^). Of the remaining 953 lean participants, 228 (23.92%) had colorectal polyps. Hemoglobin levels remained significantly higher in the participants with colorectal polyps than in those without colorectal polyps (148.8 (15.7) g/L vs. 142.5 (16.8) g/L, *P* < 0.001); thus, a positive correlation between hemoglobin levels and the prevalence of colorectal polyps was observed among lean participants. The prevalence of colorectal polyps was 15.43%, 24.24%, 30.37%, and 32.18% in quartiles 1, 2, 3, and 4, respectively (*P* for trend < 0.001). The results of this subgroup analysis show that the association of hemoglobin levels with colorectal polyps is independent of obesity.

### 3.4. Elevated Hemoglobin Levels Independently Increase the Risk of Colorectal Polyps

We also conducted stepwise multiple regression analysis to investigate the association of hemoglobin levels with the risk of colorectal polyps. Seventeen variables, including age, sex, waist circumference, BMI, systolic and diastolic blood pressure, ALT level, AST level, GGT level, triglyceride level, total cholesterol, HDL cholesterol, LDL cholesterol, fasting blood sugar, serum uric acid level, CEA level, and hemoglobin level, were input into the regression equation. We found that six variables remained in the final equation, suggesting them to be independently associated with the risk of colorectal polyps (Table 3). An important finding was that elevated hemoglobin levels were significantly associated with an increased risk of colorectal polyps (OR = 1.017; 95% CI: 1.008–1.026). This finding further supports a significant association between hemoglobin level and colorectal polyps.

## 4. Discussion

In this study, we found a significant positive association between hemoglobin levels and colorectal polyps. Hemoglobin levels were significantly higher in participants with colorectal polyps than in those without colorectal polyps. Hemoglobin levels were positively associated with the prevalence of colorectal polyps, and this association was independent of obesity. Our results also showed that elevated hemoglobin independently increased the risk of colorectal polyps. These findings may have potential clinical significance.

Currently, screening for CRC is not widely available for average-risk asymptomatic adults in many rural communities and large populations. Indeed, China serves as an example of a country with a large population and a great disparity in medical resources, and national screening programs for colorectal polyps and cancer have yet to be implemented. This delay might be attributed to the large population and the strain a screening program may place on the limited medical resources of the country [2]. Therefore, developing a more comprehensive risk assessment for asymptomatic average-risk adults may be beneficial for targeting those who are at relatively high risk and are recommended to undergo more invasive procedures, such as a colonoscopy [16].

Screening recommendations for classically described average-risk adults might be amended in areas with limited available resources; even the most effective and easy-to-use screening test, the fecal immunochemical test, may not be sufficient for detecting early adenomas [17]. Therefore, if the goal is to decrease the CRC burden, total colonoscopy is the best option for screening [5]. However, due to the increasingly aging population in China, it is almost impossible to apply the agreed-upon recommendations for screening asymptomatic adults. In addition, data on the cost-effectiveness of this strategy for these large populations are limited. One solution would be to find potential markers that may indicate the presence of colorectal polyps.

Studies have developed scoring systems that may help physicians easily stratify asymptomatic adults based on risk factors and direct them for colonoscopy [18]. This strategy might be beneficial for the identification of at-risk individuals in targeted areas and populations with limited resources as well as for scheduling of colonoscopy screening at tertiary hospitals. The application of this scheme would theoretically allow countries to increase screening and decrease the colorectal cancer burden while realistically considering the availability of medical resources.

Colorectal polyps are closely associated with metabolic diseases, such as metabolic syndrome and nonalcoholic fatty liver disease [19, 20], and several studies have reported a strong positive association of elevated hemoglobin with these conditions [9–11, 21]. This association may implicate a high hemoglobin level as a major factor in several pathologies and disorders. In this study, we focused on the association between high hemoglobin levels and colorectal polyps, and our results suggested that elevated hemoglobin may serve as an easily obtained indicator for colorectal polyps. This marker may allow the creation of a novel scoring system for predicting the presence of colorectal polyps and identifying early lesions for colonoscopy and resection.

Although the mechanism linking high hemoglobin levels and colorectal polyps is unclear, there are several probable explanations. One possible reason is that the presence of high levels of hemoglobin, an iron-containing metalloprotein, causes iron-induced oxidative stress and relative antioxidant depletion; this process has been shown to promote carcinogenesis [22, 23]. This hypothesis may support the possibility that elevated hemoglobin levels act as additive factors in the formation of colonic adenomas and their transformation into carcinomas via the adenoma-carcinoma sequence. Further clarification of the underlying mechanism will help in understanding the relationship between hemoglobin and colorectal polyps.

The strength of this study lies in the large target population of asymptomatic adults and the evaluation of different serum parameters and anthropometric measurements, which were employed to assess correlations between changes in certain parameters and the presence of colorectal polyps during screening colonoscopy. The clearly observed direct proportional association between hemoglobin level and colorectal polyps is a promising avenue for further studies to analyze the existence of a causal relationship between elevated hemoglobin and the incidence of colorectal polyps. In addition, more investigations into the pathophysiological process should be conducted.

Several limitations also exist in our study, including a lack of data regarding the dietary and smoking habits of the examined population. Possible confounding factors affecting hemoglobin levels, including smoking and increased red meat intake, might also be associated with the development of colorectal polyps [24, 25]. These confounding factors should be taken into consideration in future studies. The second limitation is that the estimated adenoma detection rate is relatively low in this study. A possible explanation is that almost half of the participants (794/1633) were younger than 50 years of age in this study. Young adults may have a lower adenoma detection rate than old ones. Moreover, histological diagnosis of the colorectal polyps found during the colonoscopies was not obtained in all cases, and different risk potentials exist for cancer progression among the different types of colorectal polyps [26]. Although we found that hemoglobin levels tended to be higher in participants with neoplastic polyps than in those with nonneoplastic polyps, further studies are necessary to confirm the association between hemoglobin levels and different types of colorectal polyps.

In conclusion, on the basis of this large colonoscopy-based study, we found a direct proportional association between hemoglobin level and colorectal polyps in an average-risk asymptomatic Chinese adult population. Further prospective studies should be performed to clarify these findings and identify any causal relationships between hemoglobin level and colorectal polyps.

## Figures and Tables

**Table 1:** Characteristics of study participants with and without colorectal polyps.

Variables	With colorectal polyps	Without colorectal polyps	*t* value	*P* value
*n* (male/female)	449 (359/90)	1184 (764/420)	36.081^a^	<0.001
Age (years)	52.1 (8.7)	49.1 (9.4)	5.925	<0.001
Waist circumference (cm)	89.1 (9.1)	85.5 (9.2)	6.993	<0.001
Body mass index (kg/m^2^)	25.09 (3.18)	24.18 (3.18)	5.157	<0.001
Systolic blood pressure (mmHg)	130.4 (16.7)	126.9 (17.7)	3.639	<0.001
Diastolic blood pressure (mmHg)	79.5 (10.8)	77.1 (11.8)	3.711	<0.001
Alanine aminotransferase (U/L)	22.0 (16.0–30.0)	19.0 (14.0–28.0)	4.171^b^	<0.001
Aspartate aminotransferase (U/L)	21.0 (18.0–25.0)	20.0 (17.0–25.0)	2.641^b^	0.008
*γ*-Glutamyltransferase (U/L)	32.0 (21.0–54.0)	25.0 (16.0–44.0)	5.647^b^	<0.001
Triglyceride (mmol/L)	1.64 (1.16–2.43)	1.42 (0.99–2.11)	4.825^b^	<0.001
Total cholesterol (mmol/L)	4.78 (4.25–5.43)	4.61 (4.08–5.24)	3.376^b^	0.001
HDL cholesterol (mmol/L)	1.13 (0.93–1.33)	1.20 (1.00–1.44)	4.058^b^	<0.001
LDL cholesterol (mmol/L)	2.75 (2.27–3.30)	2.61 (2.17–3.14)	3.169^b^	0.002
Fasting plasma glucose (mmol/L)	4.85 (4.50–5.35)	4.78 (4.41–5.19)	2.975^b^	0.003
Serum uric acid (*μ*mol/L)	366.3 (85.4)	343.1 (88.1)	4.779	<0.001
Carcinoembryonic antigen (*μ*g/L)	2.00 (1.50–2.90)	1.80 (1.30–2.60)	5.001^b^	<0.001
Hemoglobin (g/L)	151.9 (14.7)	145.9 (16.4)	6.795	<0.001

Data are expressed as the mean (SD) or median (IQR). ^a^*χ*^2^ value. ^b^*Z* value. HDL: high-density lipoprotein; LDL: low-density lipoprotein.

**Table 2:** Association between hemoglobin level and the prevalence of colorectal polyps.

Hemoglobin	Total	Colorectal polyps	PR%	PR	*χ* ^2^	*P*
Quartile 1	414	68	16.43	1.00		
Quartile 2	439	115	26.20	1.59		
Quartile 3	373	120	32.17	1.96		
Quartile 4	407	146	35.87	2.18	44.240	<0.001

PR%: prevalence rate; PR: prevalence ratio.

**Table 3:** Risk factors associated with the presence of colorectal polyps.

Variables	*β*	SE	Wald *χ*^2^	*P*	OR (95% CI)
Age (years)	0.037	0.007	29.148	<0.001	1.038 (1.024–1.052)
Waist circumference (cm)	0.020	0.008	6.651	0.010	1.020 (1.005–1.035)
Total cholesterol (mmol/L)	0.142	0.067	4.435	0.035	1.152 (1.010–1.314)
HDL cholesterol (mmol/L)	-0.476	0.204	5.445	0.020	0.622 (0.417–0.927)
Carcinoembryonic antigen (*μ*g/L)	0.094	0.033	7.947	0.005	1.099 (1.029–1.173)
Hemoglobin (g/L)	0.017	0.004	14.343	<0.001	1.017 (1.008–1.026)

*β*: partial regression coefficient; SE: standard error of the partial regression coefficient; OR: odds ratio; CI: confidence interval; HDL: high-density lipoprotein.

## Data Availability

Association between hemoglobin levels and colorectal polyps in asymptomatic Chinese adults.
